# Design and Evaluation of a MEMS Magnetic Field Sensor-Based Respiratory Monitoring and Training System for Radiotherapy

**DOI:** 10.3390/s18092742

**Published:** 2018-08-21

**Authors:** Yoonjin Oh, Young-Jin Jung, Sang Hyoun Choi, Dong Wook Kim

**Affiliations:** 1 Department of Radiation Oncology, Samsung Medical Center, Sungkyunkwan University School of Medicine, Seoul 06351, Korea; yoonjin.oh@samsung.com; 2 Department of Radiological Science, Dongseo University, Busan 47011, Korea; microbme@dongseo.ac.kr; 3 Center for Radiological Environment & Health Science, Dongseo University, Busan 47011, Korea; 4 Division of Medical Radiation Equipment, Korea Institute of Radiological and Medical Sciences, Seoul 01812, Korea; shchoi@kirams.re.kr; 5 Department of Radiation Oncology, Kyung Hee University Hospital at Gangdong, Seoul 05278, Korea

**Keywords:** radiation therapy, respiratory gating system, MEMS magnetic sensor, respiratory monitoring, respiratory training

## Abstract

The patient’s respiratory pattern and reproducibility are important factors affecting the accuracy of radiotherapy for lung cancer or liver cancer cases. Therefore, respiration training is required to induce respiration regularity before radiotherapy. However, the need for specialized personnel, space, and time-consuming training represent limitations. To solve these problems, we have developed a respiratory monitoring and training system based on a micro-electro-mechanical-system (MEMS) magnetic sensor. This system consists of a small attaching magnet, a sensor, and a breathing pattern output device. In this study, we evaluated the performance of the signal measurement in the developed system based on the various respiratory cycles, the amplitudes, and the position angles of the magnet and the sensor. The system can provide a more accurate breathing signal graph with lower measurement error and higher spatial resolution than conventional sensor methods by using additional magnet. In addition, it is possible the patient to monitor and train breathing himself by making it easy to carry and use without restriction of time and space.

## 1. Introduction

Radiation therapy has been developed to enable more accurate treatment by precise three-dimensional control of dose distribution, and leading technologies include three-dimensional conformal radiation therapy (3D-CRT), stereotactic body radiation therapy (SBRT), and intensity-modulated radiation therapy (IMRT) [1,2,3,4]. The ultimate goal of radiation therapy is to minimize the side effects that peripheral organs experience while controlling the tumor through the biological effects of radiation by fully radiating the radiation dose to the tumor site and minimizing the radiation dose in the surrounding normal tissue [5,6,7]. However, radiotherapy for tumors located in the abdominal cavity, such as lung or liver cancer, require careful consideration of the patient’s respiration because the patient’s free-breathing may cause tumor movement of greater than 2.5 cm [8,9,10,11]. During the CT scan or radiation treatment, changes in tumor location due to the patient’s breathing may increase the uncertainty in tumor targeting contouring at the treatment planning stage or target positioning at the treatment stage. Internal target volume (ITV), which represents the volume encompassing the clinical target volume (CTV) and the internal motion margin, has been proposed to reduce the uncertainty in radiotherapy that can occur from patient breathing [12,13,14,15]. However, an ITV-based treatment plan can damage the surrounding normal organs and lead to complications because of the large treatment area, which includes the extent to which tumor movement is predicted. There is a need for a method to minimize the extent of the treatment range in ITV while still transmitting the appropriate prescription dose to the tumor. Several methods have been developed to meet this need, such as respiration gating radiotherapy that irradiates only a specific phase or amplitude range in periodic respiratory motion, breath-hold radiotherapy that stops breathing during irradiation, and active-breathing control (ABC) that involves a breathing assistance device. However, these methods are not applicable to lung cancer patients who have difficulty with breathing control; in fact, the therapeutic effect may vary according to the stability and reproducibility of the patient’s breathing pattern [16,17,18,19,20,21,22].

Previous studies have shown that patients with irregular respiratory patterns can change these patterns and develop more regular patterns through respiratory training. Therefore, it is important to offer respiratory training to patients for radiation therapy [23,24,25,26]. These findings confirm that the patient’s respiratory training prior to treatment can provide more accurate radiation treatment for patients with various breathing patterns. Despite the fact that current clinical practice recommends respiratory training to produce regular breathing before treatment, this therapy has drawbacks, such as the need for support personnel and additional time and space for training.

We have developed a patient respiratory monitoring and training system based on a micro-electro-mechanical-system (MEMS) magnetic field sensor to address the clinical need for a respiratory training system that does not require additional man-power or training space. The MEMS magnetic field sensor measures the intensity of the magnetic field generated by the magnet and also measures the phase of respiratory motion to track the position of the sensor. The respiration training system based on the magnetic field sensor has a higher spatial resolution and lower noise level than the conventional method using the MEMS acceleration sensor, thus providing a high-precision breathing cycle graph to enable accurate respiratory training [27,28]. In addition, sensors, interfaces, and systems can be very small, which facilitates ease of use, as patients can carry the training system anywhere without the monitor support. In this study, we evaluated the performance of the developed MEMS magnetic field sensor-based respiratory training system by analyzing the measurement error about various respiratory cycles and amplitudes and the influence about the position change of the magnet and sensor.

## 2. Materials and Methods

### 2.1. MEMS-Based Respiratory Monitoring and Training System

**Figure 1:**
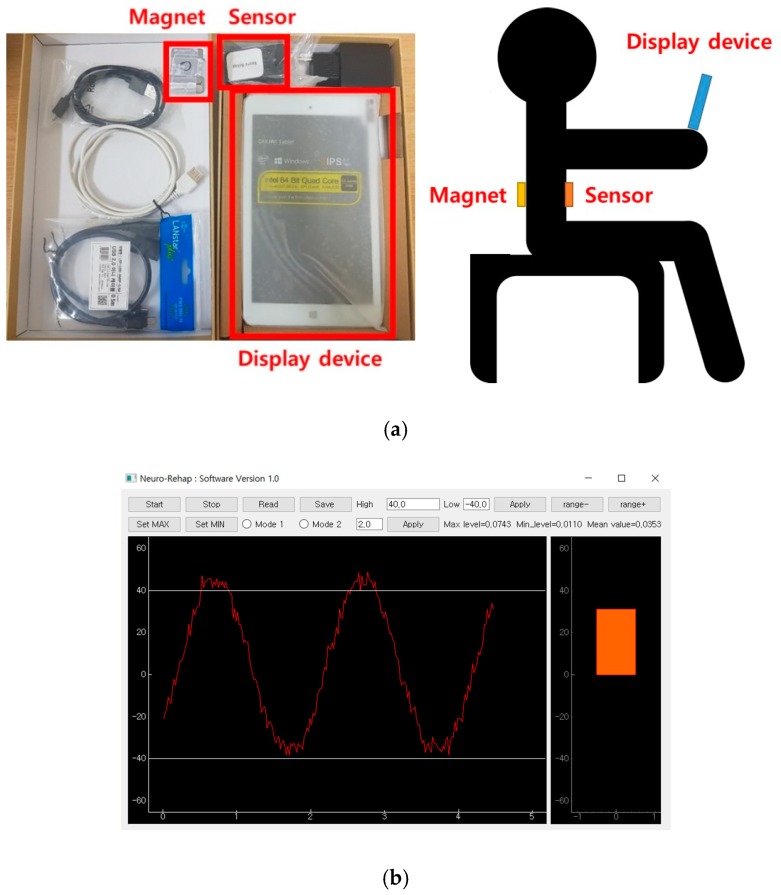
(**a**) The hardware part of the respiratory monitoring and training system; (**b**) breath pattern output device displaying the position of the sensor in a bar graph. In the graph, the *x*-axis represents time(s) and the *y*-axis represents height ratio (%) of sensor position.

The system consists of hardware and software including an attaching magnet, a sensor, and a display device, as shown in Figure 1a. The magnetic strength of the attaching magnet (emagnet, Seoul, Korea) is 3200 gauss (G) and its dimensions are 25 × 40 × 4 mm^3^. The MEMS-based magnetic field sensor (3-Space™, Portsmouth, OH, USA) has a size of 35 × 60 × 15 mm^3^, a resolution of 12 bits, a sensitivity of 0.73 mG/digit, and a scale range of −4.7–4.7 G. The sensor is located on the front of the patient’s chest, and the magnet is on the patient’s back. The sensor detects the strength of the magnetic field. The strength of the magnetic field, i.e., magnetic flux density, is inversely proportional to the distance between the sensor and the magnet, and as the distance increases, the strength of the magnetic field decreases. Therefore, the sensor estimates the position by measuring the intensity of the magnetic field according to the distance between the sensor and the magnet. The magnetic flux intensity signal detected by the sensor changes according to the movement of the chest along with the breath and is displayed on the respiratory pattern displayer through the software. The respiration pattern displaying device has a sampling rate of up to 250 Hz with a size of 35 × 60 × 15 mm^3^. The software was developed with Python 3.5 (Anaconda Python ver3.5, Austin, TX, USA) in a Windows 10 environment.

**Figure 2:**
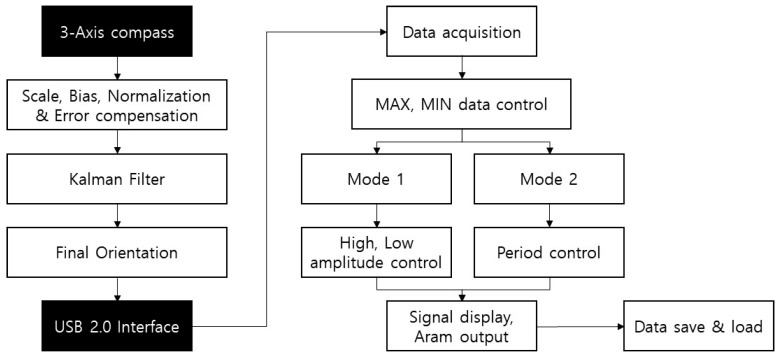
MEMS magnetic field sensor-based respiratory monitoring and training process.

The motion of the sensor attached to the patient’s chest is displayed in real time in the form of a bar graph, as shown in Figure 1b, through the respiratory pattern displaying device, so that it enables respiratory training through visual feedback. Figure 2 shows the respiratory monitoring and training process of the system. The Kalman filter is an optimal estimation filter that estimates and outputs the state variables from the measured values using a system model composed of state equations and measurement equations that represent the motion of the state variables. The signal output is obtained by a time-magnetic field strength sine wave graph along each of tri-axis, and we displayed only *y*-axis signal, which is the axis here the sensor and the magnet are located. For respiratory signal measurements, first set the maximum amplitude and minimum amplitude of the data, and select the mode for training. The training mode consists of two types, Mode 1 induces to breathe within the maximum and minimum amplitude values, and Mode 2 induces to breathe at a constant respiratory cycle. To induce respiration, the system show the patient a graph and give a reminder tone. Related information, such as the patient’s breathing pattern and time, are stored in an ASCII file for post-analysis.

### 2.2. Measurement of Respiratory Motion

The QUASAR^TM^ Programmable Respiratory Motion Phantom (Modus Medical Devices Inc., London, ON, Canada) was used to simulate various respiratory patterns for the respiratory cycle and amplitude. The QUASAR^TM^ Phantom consists of an acrylic elliptical cylindrical phantom, drive motor, and cylindrical insert with a 3-cm diameter spherical target. The QUASAR^TM^ Phantom can adjust the respiratory cycle and amplitude using the respiratory motion program. The amplitude and period of the target motion can be applied up to 3 cm and 15 s, respectively. The QUASAR^TM^ Phantom is capable of moving from a minimum of 4 cycles per minute (cpm) to a maximum of 60 cpm.

**Figure 3:**
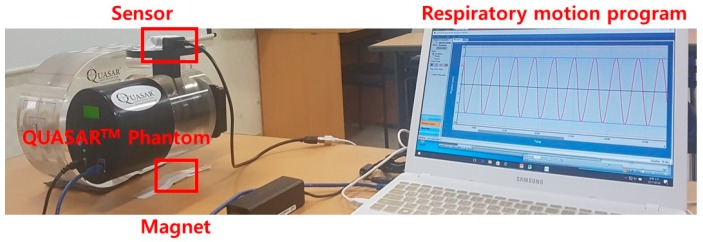
Experimental setup for measuring sensor signals with the QUASAR^TM^ Programmable Respiratory Motion Phantom and respiratory motion program.

The respiratory cycle of 1, 2, 3, 4, and 5 s and the amplitude of 1, 2, and 3 cm were chosen for measurement in consideration of the average respiratory rate of adults (Figure 3) [29]. The signal measurement time was 0.02 s. The signal acquisition time per case was 60 s on average and was measured 3 times per case. We used 58 s of signal data, excluding 2 s, to eliminate noise caused by the initial operation of the QUASAR^TM^ Phantom. The measurements were normalized to the maximum amplitude of the signal. The period and amplitude error were evaluated based on the actual phantom motion signal data output from the QUASAR^TM^ Phantom software at the given period and amplitude conditions. The measured values of the system were obtained by calculating the sine function trend equation (Equation (1)); the average period, amplitude, and standard deviation per signal were calculated and compared with the actual phantom signal:

f(x) = A*sin(Bx + C),(1)

A is the amplitude of the measured data, B is the period of the sine function, and C is the phase shift. The period of the measurement data is calculated by dividing by 2π phase. The amplitude error and the period error are obtained by subtracting the amplitude and the period of the output signal of the system from the output signal of the actual motion of the QUASARTM Phantom using Equation (1).

### 2.3. Position Dependency of Attaching Magnet

**Figure 4:**
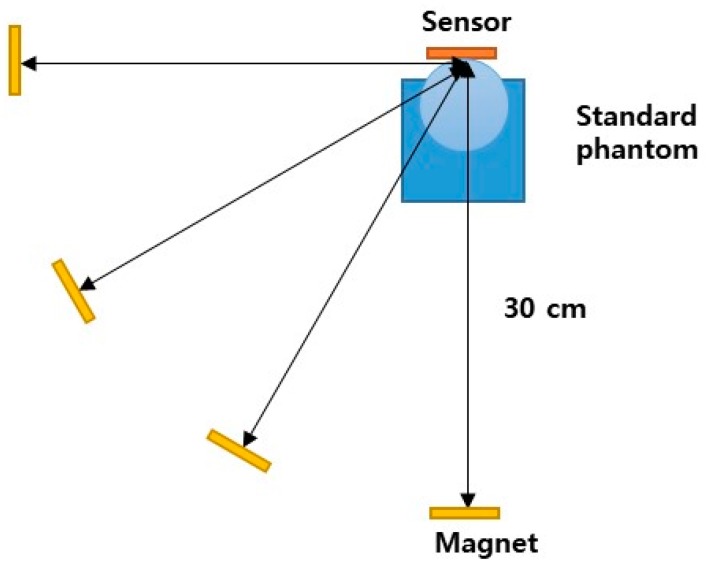
Diagram of the experimental setup for the magnet position test.

A standard respiratory motion phantom tool (GE Varian 4D solutions, Varian^®^ Medical Systems, Palo Alto, CA, USA) was used to check the influence of the position change of the magnet on signal measurement. The standard respiratory motion phantom consists of a disk that rotates at a cycle of 5.5 s, a plate that can position the IR reflector, and a drive motor. In this study, we measured the signal by changing the relative angle between the magnet and sensor for 0°, 30°, 60°, and 90° at 30 cm of distance from the sensor, as shown in Figure 4. The distance of 30 cm between the sensor and the magnet corresponds to the distance during actual breath measurement. The adult waist thickness is in range of 14.6–38.6 cm according to the human body size statistics of Korea. Amplitude error and period error are the difference between the amplitude and period of 30, 60, and 90 degrees based on 0 degree where the magnet and the sensor are in line.

## 3. Results

### 3.1. Analysis of the Respiratory Signal

**Figure 5:**
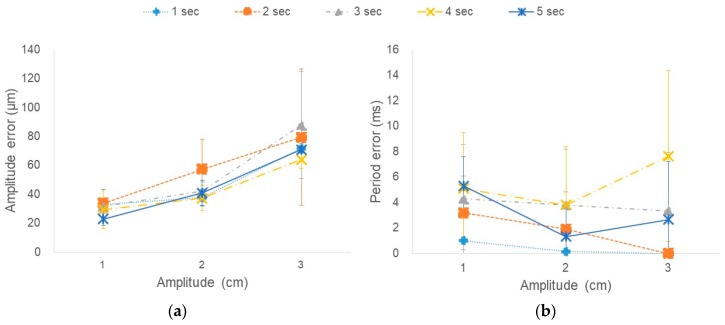
Error in (**a**) amplitude (ms) and (**b**) period (μm) by respiration pattern with a period of 1, 2, 3, 4, and 5 s and amplitude of 1, 2, and 3 cm.

As shown in Figure 5a, the amplitude error of the measured signal was lowest (22.9 μm) at 1 cm amplitude and 5 s period, and highest (87.9 μm, <0.3%) at 3 cm amplitude and 3 s period. The error of the amplitude for the 1, 2, 3, 4, and 5 s periods was 33.0, 33.8, 31.6, 29.4, and 22.9 μm for 1 cm amplitude; 37.7, 57.4, 42.1, 37.3, and 41.0 μm for 2 cm amplitude; and 71.8, 79.5, 87.9, 64.1, and 70.8 μm for 3 cm amplitude, respectively. The amplitude error increased as the set amplitude of the motion phantom became larger.

As shown in Figure 5b, the period error of the measured signal was lowest (0 ms) with a period of 1 or 2 s and highest (7.6 ms, <0.2%) with a period of 4 cm. The error of the period for the 1, 2, 3, 4, and 5 s periods was 1.0, 3.2, 4.3, 5.1, and 5.3 ms for 1 cm amplitude; 0.2, 1.9, 3.8, 3.8, and 1.3 ms for 2 cm amplitude; and 0.0, 0.0, 3.3, 7.6, and 2.6 ms for 3 cm amplitude, respectively. The error of the respiration period increased as the respiration period increased with amplitude of 1 cm.

As a result of comparing the amplitude of the respiration signal measured according to the respiration period and the amplitude set in the motion phantom, the difference between the expected and measured values showed a tendency to increase as amplitude increased and to decrease slightly as the period increased.

### 3.2. Analysis of Position Dependency of Attaching Magnet

**Figure 6:**
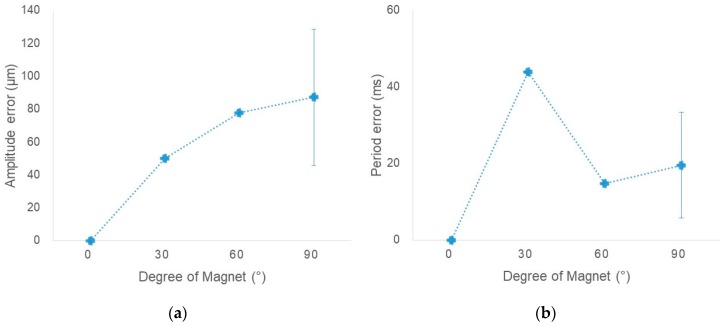
Error in (**a**) amplitude (ms) and (**b**) period (μm) by magnet position of 0°, 30°, 60°, and 90°.

The error of the measurement signal increased as the magnet angle from the sensor increased. As shown in Figure 6a, the amplitude error of the measurement signal was lowest (0 μm) when the magnet was placed in a vertical direction to the sensor and highest (87.2 μm) when it was parallel. As shown in Figure 6b, the period error of the measurement signal was lowest (0 ms) when the magnet was positioned perpendicular to the sensor. When the angle between the sensor and magnet was 0°, 30°, 60°, and 90°, the error of the measured amplitude was 0.0, 49.8, 77.7, and 87.2 μm, and the error of the measured period was 0.0, 43.9, 14.7, and 19.6 ms, respectively.

## 4. Discussion

In thoracic or abdominal radiotherapy, the patient’s respiration may cause uncertainty of delineation of the target and normal organs and lead to an unnecessary dose increase or decrease of the target and normal organs. In order to monitor such respiratory movements clinically, the techniques of taking a radiographic image and inserting an external marker or surgical node have been introduced. However, radiography and fluoroscopy are relatively costly in time, space, and money and can cause additional radiation exposure problems. Marker-based techniques can be affected by the marker attachment position and the patient’s posture. Insertion-node-based techniques require invasive methods and increased monitoring preparation time. Despite these drawbacks, adequate respiratory training for the target patient can have positive effects in most cases. In the current study, we developed and evaluated a system that self-monitors respiration without time-space constraints. Because this respiratory monitoring/training system uses magnets with a very high magnetic field, special care is required for patients using electromagnetically sensitive instruments such as pacemakers. Since the intensity of the measuring magnetic field may change according to the user’s body thickness, it is necessary to configure/calibrate for each user. To configure system for each user, the user breathes deeply to measure and set the maximum inspiratory and expiratory heights at the beginning.

The system had an error within the range of 0.7% and 0.2% for the period and amplitude of respiratory motion. The average period error was 2.9 ms, and the average amplitude error was 49.4 μm. In 2011, Ono et al. [30] reported the performance of a MEMS angular velocity sensor-based respiration monitoring system. They reported that the maximum difference of the system was 4.3%, the average periodic error was 46.7 ms, and the average amplitude error was 363.3 μm. These results are similar to reports of the respiration training system based on the MEMS acceleration sensor performed by the present researchers in 2015 [27,31,32]; respiration monitoring based on the MEMS acceleration sensor has a higher error than the MEMS magnetic field sensor. On the other hand, attempts have been made to monitor or train patient respiration with a stereo vision camera. In 2018, Bae et al. evaluated the performance of a stereo vision-based respiratory monitoring system and reported that it had an amplitude error of 2.3% to 16.3% and an average amplitude error of 800 μm. [33]. Using a Kinect v2 camera, Silverstein et al. developed a system for monitoring respiration using information on the surface of the patient without markers and compared their system with existing commercial products, Varian’s Real-time Position Management (RPM) System (Varian Medical Systems, Palo Alto, CA, USA) and the Anzai belt system (Anzai Medical Co., Ltd., Tokyo, Japan). They reported that the period measurement error of their system was 77 ms to 164 ms [34]. According to the accuracy test of the RPM system used for respiratory monitoring in the present clinical cases, 99.9% of cases have an error of less than 2 mm [35]. In 2015, Massaroni et al. evaluated the performance of the opto-electronic plethysmography (OEP) using eight markers and reported that it had a 3D relative motion discrepancy of −64 to 76 μm [36]. They reported that the breathing rate error of the fiber Bragg grating (FBG) sensor was −45 to 145 ms using 42 markers [37]. Our system has similar performance to the results of using these multiple markers and is convenient for use during radiation therapy because only one marker is used. Considering the results of previous studies, the performance of the MEMS magnetic sensor-based respiration training system was comparable or better than other existing systems.

In 2008, Venkat et al. [25] reported that period and amplitude changes can be reduced by about half by appropriate respiratory training. In this way, the MEMS magnetic field sensor-based respiration training system could be used to stabilize unstable breathing patterns by providing a training mode that induces a constant breathing amplitude and cycle. Also, since the system had an average period error of 19.5 ms and amplitude error of 53.7 μm depending on the magnet position, there was not much difference in signal measurement according to the position of the magnet and sensor. This finding indicates that there was little dependence on the user’s position.

These results suggest that our system based on the MEMS magnetic field sensor could increase the accuracy of respiration monitoring, as the system has less measurement error for respiratory motion than previous technologies. Moreover, the system is easy to operate and easy to use anytime and anywhere, and it enables patients to monitor and train their own breathing, which is expected to shorten radiotherapy time and improve treatment results. In this study, further review of system responses to long-term use are needed, given that patients require long-term breathing training in general clinical situations.

## 5. Conclusions

In this study, we evaluated the performance of a respiratory monitoring and training system based on the MEMS magnetic field sensor developed to correct the pattern and reproducibility of respiratory motion in patients and thus increase the accuracy of radiotherapy. The respiration signal period and amplitude error of the system were 0 ms to 7.6 ms and 22.9 μm to 87.9 μm, respectively, and there was a maximum difference of 43.9 ms and 87.2 μm depending on the measurement position. These findings indicate that breathing signal measurement was stable and patient breath monitoring was possible in various postures. The system is easy to carry with this performance and can be used by the patients themselves without limitations of time, space and monitoring support. If this system is used for respiration training, it will contribute to the improvement of radiation therapy outcomes by helping to maintain respiration stability and reproducibility during radiotherapy.
